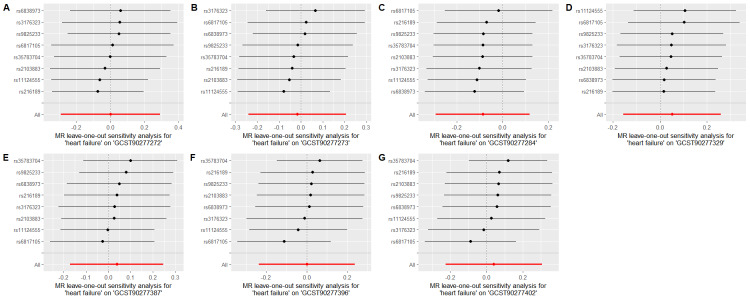# Immune cell-mediated effects of plasma lipids on heart failure: A two-step, two-sample Mendelian randomization study

**DOI:** 10.1097/MD.0000000000049074

**Published:** 2026-05-29

**Authors:** Zonglian Liu, Eugene Kwaku, Yangyang Cui, Lijuan Xiong, Shikang Li, Lang Zeng, Linqin Du, Kun Wang, Jiankang Zheng, Dalin Luo, Gang Zeng, Rongchuan Yue

**Affiliations:** a Department of Cardiology, Zizhong People’s Hospital, Neijiang, Sichuan Province, P.R. China; b Department of Cardiology, Affiliated Hospital of North Sichuan Medical College, Nanchong, Sichuan Province, P.R. China; c Department of Cardiology, People’s Hospital of Guang’an District, Guang’an, Sichuan Province, P.R. China.

**Keywords:** causality, heart failure, immune cells, mediating effects, Mendelian randomization, plasma lipids

## Abstract

A growing number of studies have shown that plasma lipids and immune cells are related to the occurrence and development of heart failure (HF), but the causal relationship between them is unclear. Therefore, it is critical to obtain a thorough understanding of the impact of plasma lipids and immune cells on the risk of developing HF. In addition, it is important to further explore whether immune cells play a mediating role in this relationship. First, a two-way, two-sample Mendelian randomization (MR) analysis was performed on 179 plasma lipids and HF, and the effect size (β) was obtained. Then, MR analysis was conducted on 731 kinds of immune cells in relation to HF. Moreover, the MR analysis of the screened plasma lipids in relation to immune cells produced an effect value of β1, and the MR analysis of immune cells causally related to plasma lipids with HF yielded an effect value of β2. Finally, the proportion of mediated effects of immune cells between plasma lipids and HF was calculated by the formula β1 × β2/β. During the study, we employed several analytical methods, including inverse-variance weighting, Bayesian weighted Mendelian randomization, MR-Egger regression, weighted median, weighted model, and simple model. Moreover, heterogeneity and pleiotropy tests were employed to validate the results. The results indicated a causal relationship between 7 types of plasma lipids and HF, and 4 types of immune cells mediated the causal relationship between plasma lipids and HF. The proportion of mediated effects in which HLA-DR+ CD4+ AC-mediated triacylglycerol (50:1) levels affecting HF was 5.77%. The results of this study were confirmed to be reliable by heterogeneity testing and sensitivity analysis. There was a causal relationship between 7 types of plasma lipids and HF, and 4 types of immune cells mediated the causal relationship between lipids and HF. This finding is expected to provide new ideas for the therapeutic strategy of HF.

## 1. Introduction

In the world, cardiovascular diseases (CVDs) continue to be the leading cause of death. According to statistics, in 2021 alone, 20.5 million people died from CVD, accounting for about 33.3% of all deaths worldwide.^[1]^ Common CVDs include coronary artery disease, hypertension, heart failure (HF), and arrhythmia, among others. HF is frequently the final stage of CVD, characterized by high morbidity and mortality rates. It has become a major public health and clinical problem worldwide.^[2–4]^ According to epidemiological surveys, the prevalence of HF in adults is 1% to 3% around the world,^[4]^ the standardized prevalence of HF in China is 1.1%, with an incidence rate of 275 per 100,000 people,^[5]^ which has placed an increasing burden on health systems and seriously damaged people’s health. Therefore, it is essential to actively search for potential risk factors for HF to provide new targets for the prevention and treatment of HF.

Lipids are essential structural components of the cell membranes^[6]^ and are closely associated with the occurrence and development of CVD, such as atherosclerosis, HF, myocardial infarction, and so on.^[7–9]^ The plasma lipidome refers to a comprehensive analysis of individual lipids within biological systems.^[10]^ In Ottosson’s study, the plasma lipid profile offers a more comprehensive characterization of dyslipidemia, which can be predicted up to 20 years prior to the onset of CVD.^[11]^ Several studies have demonstrated that the plasma lipidome plays a significant role in the onset and progression of HF.^[12–14]^ However, HF is a complex systemic disease, and its occurrence and development are influenced not only by plasma lipid levels but also by activation of the immune system and inflammatory response.^[15]^ Studies have demonstrated that both innate and adaptive immunity are activated in the context of HF.^[16]^ Inflammation is increasingly recognized as a critical factor in the development of CVD.^[17]^ Therefore, it is critical to gain a comprehensive understanding of the impact of plasma lipids and immune cells on the risk of developing HF. In addition, further exploration is needed to explore whether immune cells play a mediating role in this relationship.

To mitigate the impact of various unfavorable factors such as reverse causality and confounding variables, which are inherent in traditional research methods, Mendelian randomization (MR) was used for the analysis of this study. This method uses genetic variation as an instrumental variable (IV) to determine the relationship between exposure and outcome.^[18]^ In this method, genetic variants, specifically, single-nucleotide polymorphisms (SNPs), are used as IVs that are randomly assigned to minimize the effects of confounding factors and reverse causality, so it is similar to a randomized clinical trial.^[19]^ Compared with a randomized clinical trial, MR research provides several advantages, including ease of handling, lower cost, and greater efficiency. The emergence and development of genomics research have ushered in a new era in CVD research, enabling researchers to delve deeper into the genetic and molecular mechanisms behind HF.^[20]^ Current MR studies support the causal relationship between plasma lipids, immune cells, and HF.^[21,22]^ But there is still a lack of research on whether immune cell subsets affect the causal relationship between plasma lipids and HF. To fill this gap, this study conducted a mediated MR study using publicly available genome-wide association study (GWAS) data, which is expected to obtain potential genetic targets related to HF, thereby providing new insights into the pathogenesis of HF. This will provide robust support for future treatment and prevention strategies for HF.

## 2. Materials and methods

### 2.1. Study design

**Figure 1.**
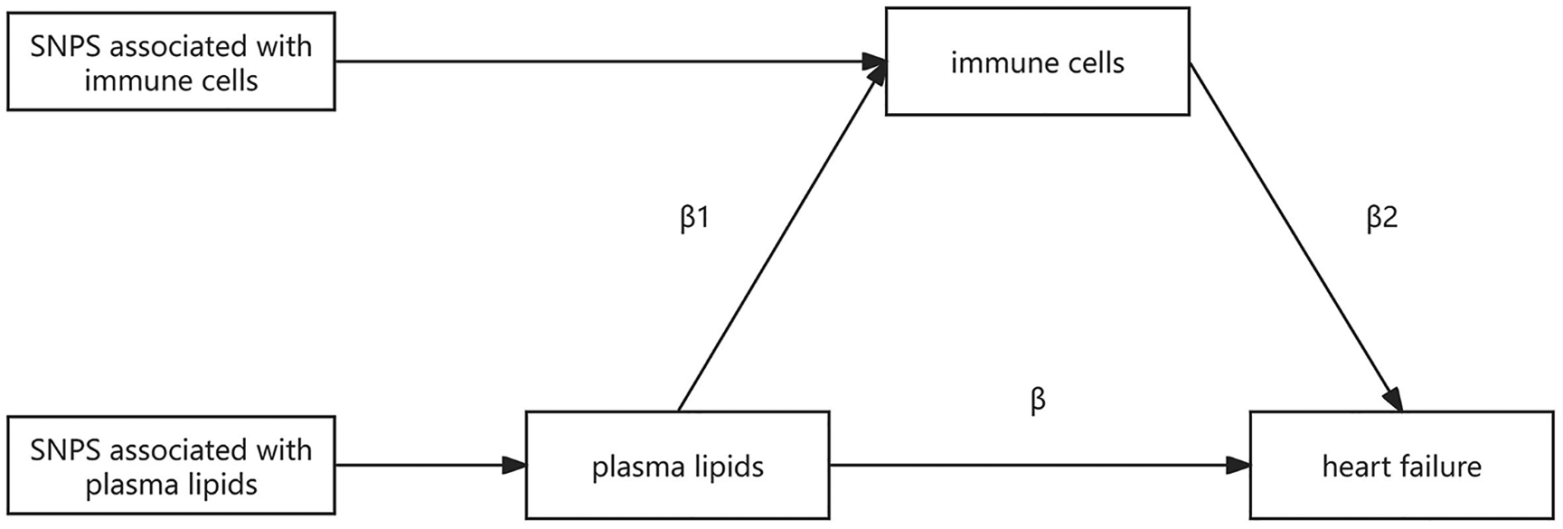
Two-step Mendelian randomization study of immune cell-mediated plasma lipids on heart failure. SNP = single-nucleotide polymorphism.

This study was based on a two-sample MR methodology that strictly enforced the 3 core assumptions of MR: the assumption of correlation: there is a strong association between the IVs and the exposure studied; the assumption of independence: the IVs have no relationship to exposure-outcome confounders; the assumption of exclusivity: the IVs can only affect the outcome through the exposure. First, a two-way, two-sample MR analysis was conducted on 179 plasma lipids in relation to HF, and the effect value β was obtained. Then, MR analysis was performed on 731 kinds of immune cells and HF, Moreover, MR analysis of the screened plasma lipids with immune cells yielded an effect value of β1, and MR analysis of immune cells causally related to plasma lipids with HF yielded an effect value of β2; finally, the proportion of mediated effects of immune cells between plasma lipids and HF was calculated by the formula β1 × β2/β. The study design is shown in Figure 1. The data utilized in this MR study are publicly available; therefore, this paper is exempt from informed consent and ethical approval.

### 2.2. Data source

The individuals included in this MR analysis were all from European populations. GWAS summary data for both plasma lipids and immune cells were obtained from the GWAS Catalog database (https://www.ebi.ac.uk/gwas/). The GWAS summary data for 179 plasma lipids are from the study by Ottensmann et al.^[10]^ Its registration number is GCST90277238-GCST90277416, which includes a total of 7174 Finns for analysis. The GWAS summary data for 731 immune cells are from the study by Orru et al.^[23]^ Its registration number is GCST0001391-GCST0002121, which includes 3757 Sardinians and tested approximately 22 million SNPs after adjusting for sex and age. Summary data from a GWAS on HF were obtained from the Finnish database (https://r11.finngen.fi/). Its registration number is finngen_R11_I9_HEARTFAIL, which includes a sample of 453,733 participants for analysis. Of these, 33,250 were in the case group, and 420,483 were in the control group, with a total of 21,306,794 SNPs.

### 2.3. Screen for relevant IVs

To improve the accuracy and reliability of the analysis, it is necessary to include as many IVs as possible to improve the explanatory power regarding the exposure factors.^[24]^ Therefore, when the SNPs of plasma lipids and immune cells were used as IVs, the significance threshold of *P* < 5 × 10^−6^ was applied for screening. SNPs of HF were screened with a significance threshold of *P* < 5 × 10^−8^ when considered as IVs. The entire study was conducted with a linkage disequilibrium measure *r*^2^ = 0.001 and a region width of kb = 10,000, thus ensuring that the SNPs were independent of each other. The formula *F* = [*R*^2^ × (N − 1 − *K*)]/[*K* × (1 − *R*^2^)] was utilized, where *R*^2^ = 2 × MAF × (1 − MAF) × β^2^, to calculate the *F* value of each SNP. Weak IVs with *F* < 10 were excluded to ensure a strong correlation between IVs and exposure.^[25]^ Then remove registry numbers with <3 SNPs (GCST0001563, GCST0001714, GCST0001764, GCST0001895, GCST0002092, and GCST0002107). Finally, during the process of integrating the exposure and outcome data, the SNPs significantly related to the outcome were removed, and the incompatible and nondirectional palindromic SNPs were removed successively.

### 2.4. Statistical analysis

The analyses in this study were based on several program packages in R 4.3.2 software (R Foundation for Statistical Computing), including “TwoSampleMR (version 0.5.8), MR-PRESSO (version 1.0), MRInstruments (version 0.3.2), MendelianRandomization (version 0.9.0), BWMR (version 0.1.1)” and several other program packages. The significance level is set at α = 0.05. Inverse-variance weighted (IVW) analysis was used as the primary analysis, with additional analyses using the Bayesian weighted Mendelian randomization (BWMR) method, MR-Egger regression, weighted median method, weighted model method, and simple model method. The odds ratio (OR) and 95% confidence interval (CI) were used to evaluate the causal relationship between exposure and outcome.

### 2.5. Sensitivity analysis

First, the Cochran *Q* test was used to assess the presence of heterogeneity among SNPs,^[26]^ and the formula *I*^2^ = (*Q* − df)/*Q* × 100% was also utilized to calculate the *I*^2^ statistic to reflect the heterogeneous portion of genetic variation as a proportion of the total variation. If *I*^2^ = 0, we considered that there was no heterogeneity in the study, when 0 < *I*^2^ < 25%, 25% < *I*^2^ < 50%, and *I*^2^ > 50%, it indicated that there was low, medium, and high heterogeneity in the MR study, if *I*^2^ > 50%, it indicated that there was a high degree of heterogeneity and that a random-effects model needed to be used.^[27–29]^ The presence or absence of horizontal pleiotropy among SNPs was assessed using the MR-Egger intercept method,^[30]^ and MR-PRESSO was used to detect the presence or absence of outliers and to assess further the presence or absence of horizontal pleiotropy among SNPs.^[31]^ Second, the leave-one-out (LOO) method was employed to assess the robustness and reliability of the causal relationship between exposure and outcome.^[32]^ Finally, scatter plots were drawn to visualize the results of the MR analysis.

## 3. Results

### 3.1. MR analysis of plasma lipids and HF

**Figure 2.**
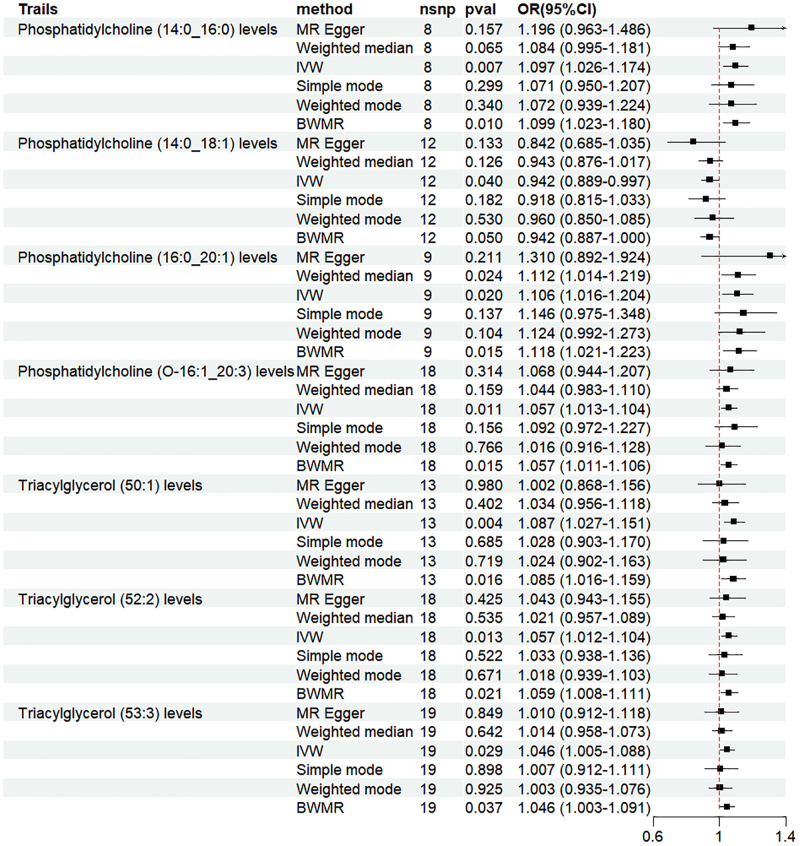
Forest plot of the results of MR and BWMR analysis between plasma lipids and heart failure. BWMR = Bayesian weighted Mendelian randomization, CI = confidence interval, MR = Mendelian randomization, OR = odds ratio.

The MR analysis was conducted under specific screening conditions. When the β values of the 5 analysis methods were in the same direction, the IVW results were mainly concerned.^[33]^ The IVW results showed: phosphatidylcholine (14:0:0) levels (OR = 1.097, 95% CI = 1.026–1.174, *P* = .007), phosphatidylcholine (16:0_20:1) levels (OR = 1.106, 95% CI = 1.016–1.204, *P* = .020), phosphatidylcholine (O-16:1:3) levels (OR = 1.057, 95% CI = 1.013–1.104, *P* = .011), triacylglycerol (50:1) levels (OR = 1.087, 95% CI = 1.027–1.151, *P* = .004), triacylglycerol (52:2) levels (OR = 1.057, 95% CI = 1.012–1.104, *P* = .013), and triacylglycerol (53:3) levels (OR = 1.046, 95% CI = 1.005–1.088, *P* = .029) were positively correlated with HF. Phosphatidylcholine (14:0_18:1) levels (OR = 0.942, 95% CI = 0.889–0.997, *P* = .040) showed a negative causal relationship with HF. Analysis by the BWMR method yielded results consistent with IVW, as illustrated in Figures 2 and S1, Supplemental Digital Content.

### 3.2. Sensitivity analysis of plasma lipids and HF

Cochran *Q* test showed that there was no statistical heterogeneity detected among the selected SNPs (*P* > .05); the calculated *I*^2^ statistics ranged from 0% to 40.2%, and most of them were 0, indicating that the percentage of heterogeneity that existed was very low, as shown in Table S1, Supplemental Digital Content. MR-PRESSO did not detect any outliers, and the *P* values of its MR analysis results were consistent with those of the IVW analysis results (*P* < .05), reinforcing the robustness and reliability of the IVW results; the MR-Egger intercept method and MR-PRESSO suggested the absence of horizontal pleiotropy (*P* > .05), as shown in Table S2, Supplemental Digital Content. The LOO method results showed that no individual SNPs significantly affected the outcome, suggesting that the results of the MR analyses were robust, as illustrated in Figure S2, Supplemental Digital Content.

### 3.3. MR analysis of HF and plasma lipids

**Figure 3.**
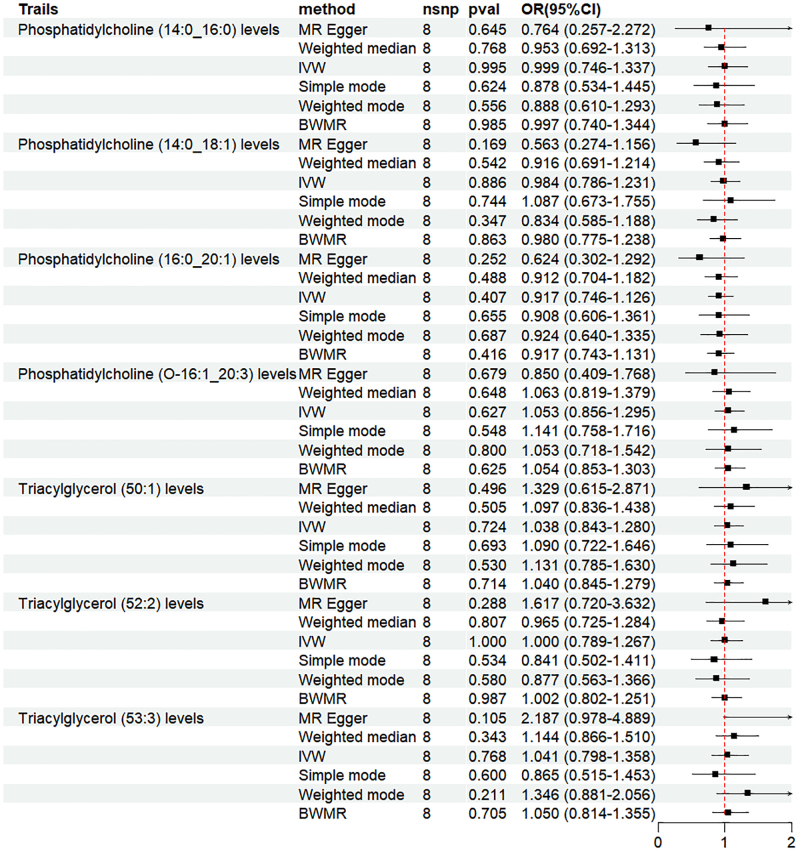
Forest plot of the results of MR and BWMR analysis between heart failure and plasma lipid groups. BWMR = Bayesian weighted Mendelian randomization, CI = confidence interval, MR = Mendelian randomization, OR = odds ratio.

To determine whether reverse causality existed in this association, HF was treated as the exposure, while the 7 plasma lipids identified in the forward MR analysis were analyzed as the outcome by reverse MR analysis. The MR analysis indicated that there was no evidence of reverse causality between the variables (*P* > .05). The results consistent with IVW were obtained using the BWMR analysis, as shown in Figures 3 and S3, Supplemental Digital Content.

### 3.4. Sensitivity analysis of HF and plasma lipids

The results of the Cochran *Q* test showed that there was no statistical heterogeneity detected among the selected SNPs (*P* > .05); the calculated *I*^2^ statistic ranged from 0% to 42.2%, suggesting that a certain percentage of heterogeneity existed, as shown in Table S3, Supplemental Digital Content. MR-PRESSO did not reveal any outliers; the MR-Egger intercept method and MR-PRESSO suggested that there was no horizontal multiplicity of validity (*P* > .05), as shown in Table S4, Supplemental Digital Content. The results of the LOO method showed that no single SNP significantly affected the outcome, suggesting that the MR analysis was robust, as illustrated in Figure S4, Supplemental Digital Content.

### 3.5. MR analysis of immune cells and HF

**Figure 4.**
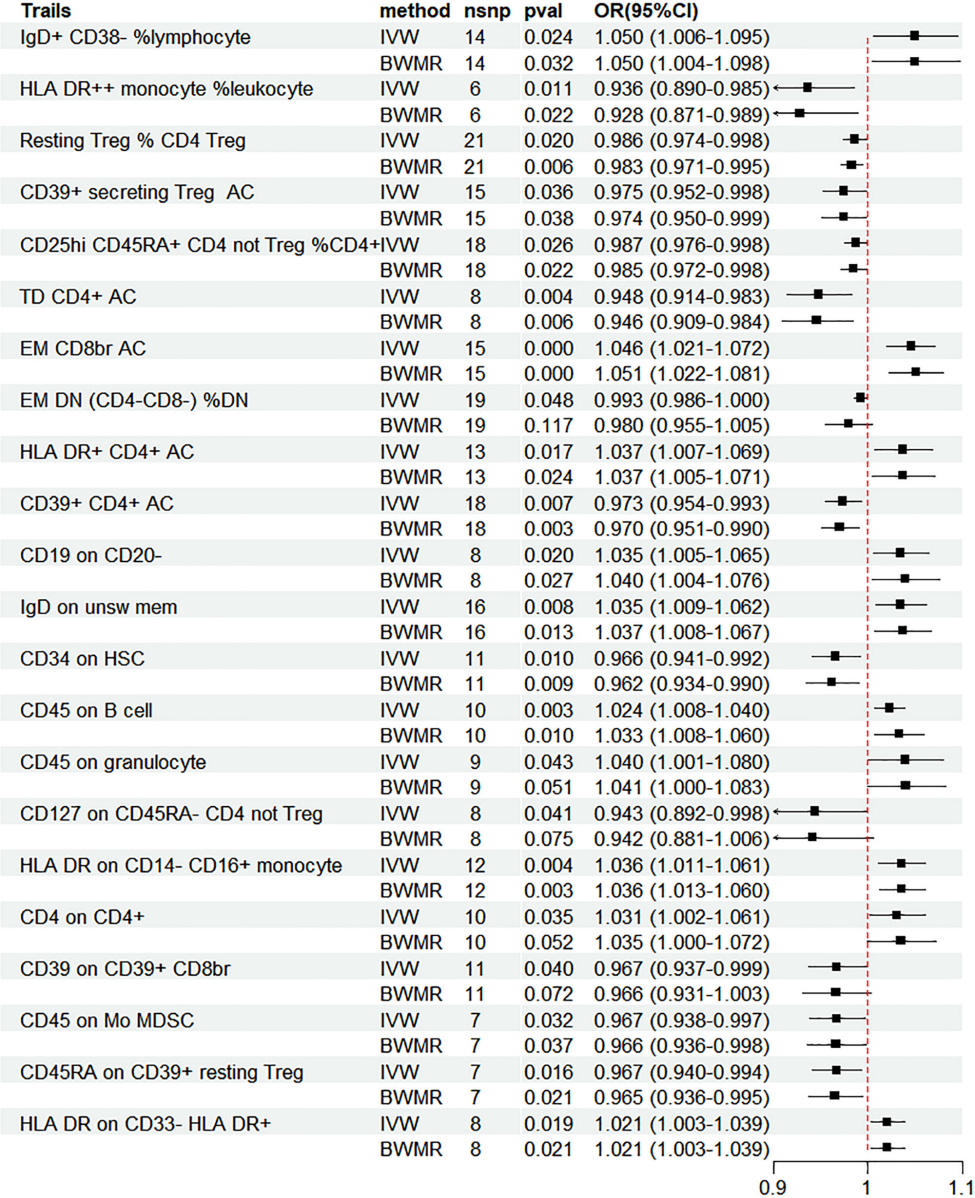
Forest plot of the results of MR and BWMR analysis between immune cells and heart failure. BWMR = Bayesian weighted Mendelian randomization, CI = confidence interval, MR = Mendelian randomization, OR = odds ratio.

MR analysis was performed between 731 immune cells and HF, and 22 immune cells were associated with HF. The IVW results showed: IgD+ CD38− %lymphocyte (OR = 1.050, 95% CI = 1.006–1.095, *P* = .024), EM CD8br AC (OR = 1.046, 95% CI = 1.021–1.072, *P*<.001), HLA-DR+ CD4+ AC (OR = 1.037, 95% CI = 1.007–1.069, *P* = .017), CD19 on CD20− (OR = 1.035, 95% CI = 1.005–1.065, *P* = .020), IgD on unsw mem (OR = 1.035, 95% CI = 1.009–1.062, *P* = .008), CD45 on B cell (OR = 1.024, 95% CI = 1.008–1.040, *P* = .003), CD45 on granulocyte (OR = 1.040, 95% CI = 1.001–1.080, *P* = .042), HLA-DR on CD14− CD16+ monocyte (OR = 1.036, 95% CI = 1.011–1.061, *P* = .004), CD4 on CD4+ (OR = 1.031, 95% CI = 1.002–1.061, *P* = .035), and HLA-DR on CD33− HLA DR+ (OR = 1.021, 95% CI = 1.026–1.1003, *P* = .019) had a positive causal relationship with HF; but HLA-DR++ monocyte %leukocyte (OR = 0.936, 95% CI = 0.890–0.985, *P* = .011), resting Treg %CD4 Treg (OR = 0.986, 95% CI = 0.974–0.998, *P* = .020), CD39+ secreting Treg AC (OR = 0.975, 95% CI = 0.952–0.998, *P* = .036), CD25hi CD45RA+ CD4 not Treg %CD4+ (OR = 0.987, 95% CI = 0.976–0.998, *P* = .026), TD CD4+ AC (OR = 0.948, 95% CI = 0.914–0.983, *P* = .004), EM DN (CD4−CD8−) %DN (OR = 0.993, 95% CI = 0.986–1.000, *P* = .047), CD39+ CD4+ AC (OR = 0.973, 95% CI = 0.954–0.993, *P* = .007), CD34 on HSC (OR = 0.966, 95% CI = 0.941–0.992, *P* = .010), CD127 on CD45RA− CD4 not Treg (OR = 0.943, 95% CI = 0.892–0.998, *P* = .041), CD39 on CD39+ CD8br (OR = 0.967, 95% CI = 0.937–0.999, *P* = .040), CD45 on Mo MDSC (OR = 0.967, 95% CI = 0.938–0.997, *P* = .032), and CD45RA on CD39+ resting Treg (OR = 0.967, 95% CI = 0.940–0.994, *P* = .016) showed a negative causal relationship with HF. BWMR analysis was also performed, as shown in Figures 4 and S5, Supplemental Digital Content.

### 3.6. Sensitivity analysis of immune cells and HF

The results of the Cochran *Q* test showed that there was no statistical heterogeneity detected among the selected SNPs (*P* > .05); the calculated *I*^2^ statistic ranged from 0% to 30.5%, and most of the *I*^2^ statistic was 0, suggesting that a low percentage of heterogeneity existed, as shown in Table S5, Supplemental Digital Content. MR-PRESSO did not detect any outliers; the MR-Egger intercept method, the MR-PRESSO suggests that there was no presence of horizontal pleiotropy (*P* > .05), as shown in Table S6, Supplemental Digital Content. The results of the LOO method showed that no single SNP significantly influenced the outcome, suggesting that the findings of the MR analyses were robust, as illustrated in Figure S6, Supplemental Digital Content.

### 3.7. Mediator MR analysis

**Figure 5.**
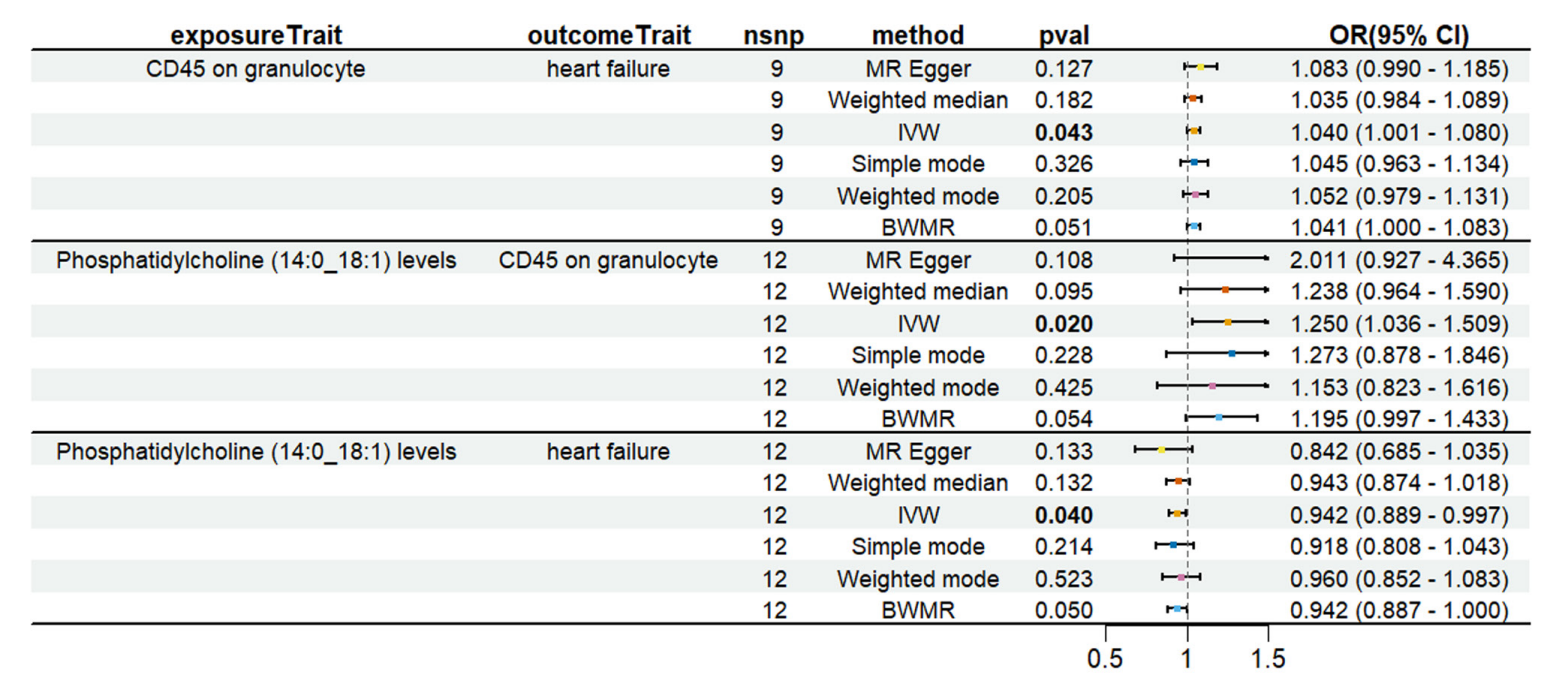
CD45 on granulocyte mediated analysis between phosphatidylcholine (14:0_18:1) levels and heart failure. BWMR = Bayesian weighted Mendelian randomization, CI = confidence interval, IVW = inverse-variance weighted, MR = Mendelian randomization, OR = odds ratio, SNP = single-nucleotide polymorphism.

**Figure 6.**
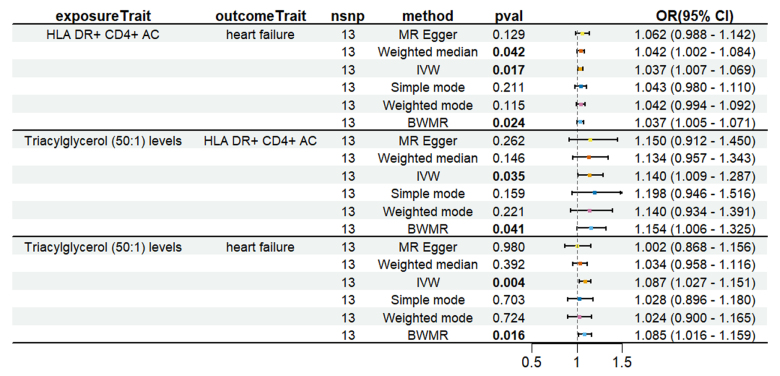
HLA-DR+ CD4+ AC-mediated analysis between triacylglycerol (50:1) levels and heart failure. BWMR = Bayesian weighted Mendelian randomization, CI = confidence interval, IVW = inverse-variance weighted, MR = Mendelian randomization, OR = odds ratio, SNP = single-nucleotide polymorphism.

**Figure 7.**
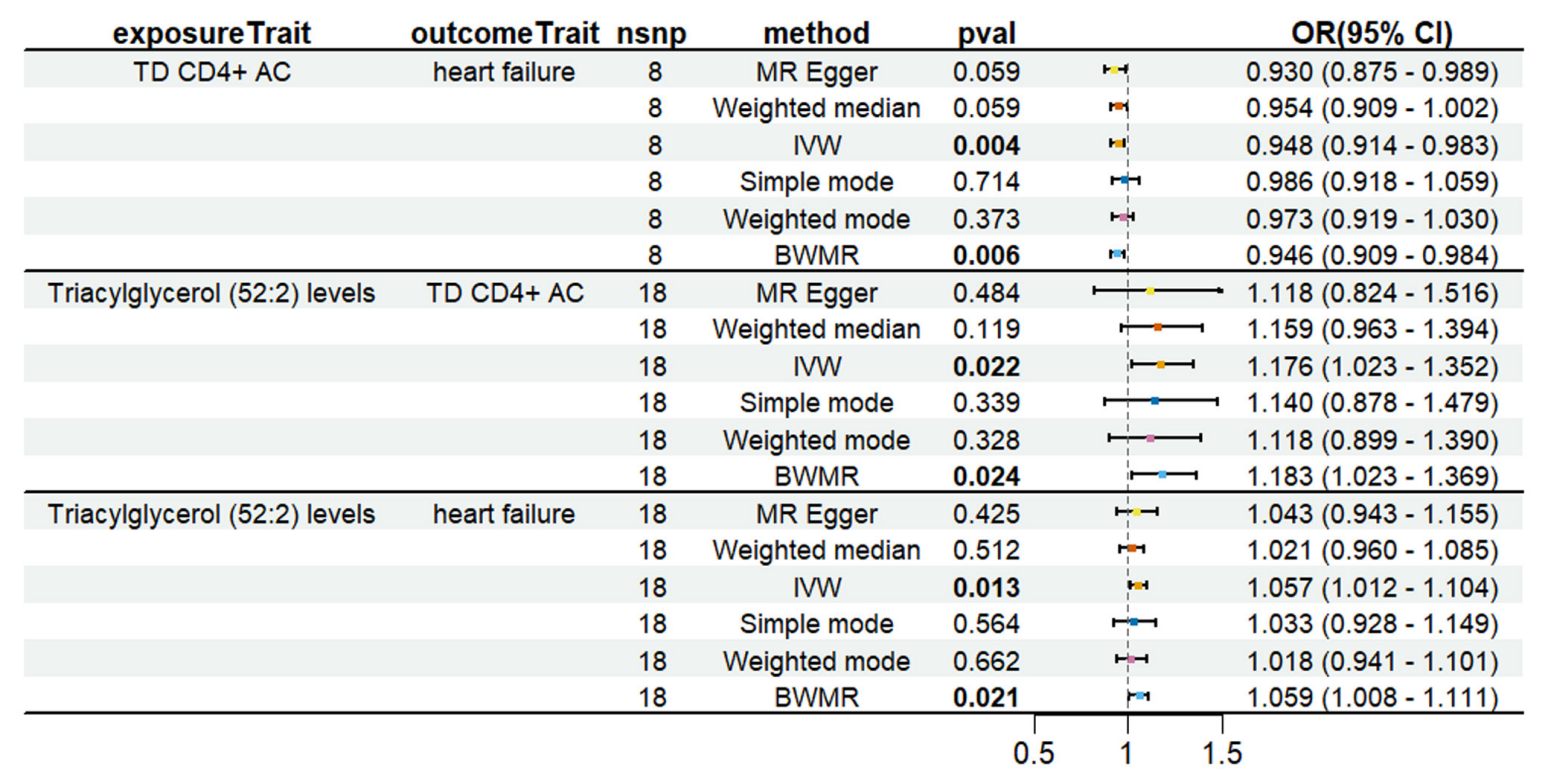
TD CD4+ AC-mediated analysis between triacylglycerol (52:2) levels and heart failure. BWMR = Bayesian weighted Mendelian randomization, CI = confidence interval, IVW = inverse-variance weighted, MR = Mendelian randomization, OR = odds ratio, SNP = single-nucleotide polymorphism.

**Figure 8.**
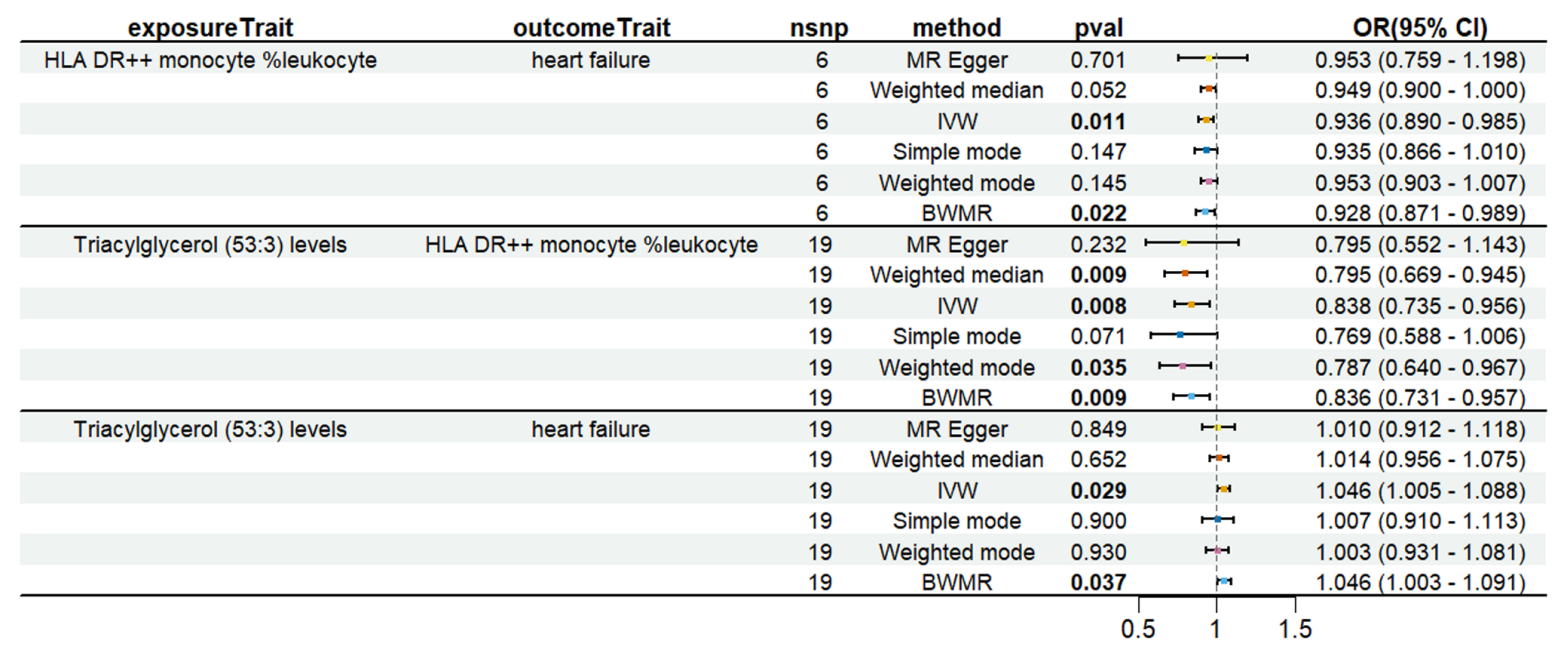
HLA-DR++ monocyte %leukocyte mediated analysis between triacylglycerol (53:3) levels and heart failure. BWMR = Bayesian weighted Mendelian randomization, CI = confidence interval, IVW = inverse-variance weighted, MR = Mendelian randomization, OR = odds ratio, SNP = single-nucleotide polymorphism.

To evaluate whether immune cells act as a mediator in influencing the causal relationship between plasma lipids and HF, the 7 kinds of plasma lipids and 22 kinds of immune cells screened were analyzed by MR, and ensured that the OR values of the 5 analytical methods were in the same direction, after Cochran *Q* test (*P* > .05), MR-PRESSO, MR-Egger intercept method (*P* > .05), and LOO method analysis, the IVW results showed that phosphatidylcholine (14:0_18:1) levels were associated with CD45 on granulocyte (OR = 1.250, 95% CI = 1.036–1.509, *P* = .020), triacylglycerol (50:1) levels with HLA-DR+ CD4+ AC (OR = 1.140, 95% CI = 1.009–1.287, *P* = .035), triacylglycerol (52:2) levels with TD CD4+ AC (OR = 1.176, 95% CI = 1.023–1.352, *P* = .022), triacylglycerol (53:3) levels with HLA-DR++ monocyte %leukocyte (OR = 0.838, 95% CI = 0.735–0.956, *P* = .008), and yielded an effect value of β1. Then the 4 kinds of immune cells causally associated with plasma lipids and HF were analyzed by MR, again after the Cochran *Q* test (*P* > .05), MR-PRESSO, and MR-Egger intercept method (*P* > .05), the results of their IVW showed: HLA DR++ monocyte %leukocyte (OR = 0.936, 95% CI = 0.890–0.985, *P* = .011), TD CD4+ AC (OR = 0.948, 95% CI = 0.914–0.983, *P* = .004), HLA-DR+ CD4+ AC (OR = 1.037, 95% CI = 1.007–1.069, *P* = .017), CD45 on granulocyte (OR = 1.040, 95% CI = 1.001–1.080, *P* = .043), yielded an effect value β2 (Figs. 5–8). The results of heterogeneity analysis and multiplicity analysis conducted in this session are presented in Tables S7 and S8, Supplemental Digital Content.

### 3.8. Mediator effect analysis

In conclusion, we identified a causal relationship between 4 immune cell-mediated plasma lipids and HF. The effect value β for triacylglycerol (50:1) levels in the occurrence of HF was 0.084, the effect value β1 for HLA-DR+ CD4+ AC was 0.131, while the effect value β2 for HLA-DR+ CD4+ AC in the occurrence of HF was 0.037, the proportion of their mediator effect was β1 × β2/β = 5.77%. While the remaining 3 mediator relationships were not calculated for the mediator effect ratios because their β, β1, and β2 were not the same direction, the specifics were the effect value β for phosphatidylcholine (14:0_18:1) levels in the occurrence of HF β was −0.060, the effect value β1 in the occurrence of CD45 on granulocyte β was 0.131, and the effect value β2 in the occurrence of CD45 on granulocyte with HF was 0.223; the effect value β for triacylglycerol (52:2) levels in the occurrence of HF was 0.055, the effect value β1 in the occurrence of TD CD4+ AC was 0.162, and the effect value β2 in the occurrence of TD CD4+ AC in HF was −0.054; the effect value β for triacylglycerol (53:3) levels in the occurrence of HF was 0.045, the effect value β1 in the occurrence of HLA-DR++ monocyte %leukocyte was −0.176, and the effect value β2 in the occurrence of HLA-DR++ monocyte %leukocyte in HF was −0.066.

## 4. Discussion

To our knowledge, this is the first instance in which a bidirectional, two-sample, two-step MR approach has been used to investigate the causal relationship between 179 plasma lipids and HF and whether 731 immune cells act as potential mediators. Our results showed that phosphatidylcholine (14:0_16:0) levels, phosphatidylcholine (16:0_20:1) levels, phosphatidylcholine (O-16:1_20:3) levels, triacylglycerol (50:1) levels, triacylglycerol (52:2) levels, and triacylglycerol (53:3) levels showed a positive causal relationship with HF. In contrast, phosphatidylcholine (14:0_18:1) levels showed a negative causal relationship with HF. Subsequently, we conducted a two-step MR analysis to explore whether immune cells mediate the relationship between plasma lipids on the risk of developing HF. Ultimately, we found that HLA-DR+ CD4+ AC mediated the association between triacylglycerol (50:1) levels and the risk of developing HF, with a mediating effect ratio of 5.77%, while CD45 on granulocyte on phosphatidylcholine (14:0_18:1) levels, TD CD4+ AC on triacylglycerol (52:2) levels, HLA-DR++ monocyte %leukocyte on triacylglycerol (53:3) levels and the risk of developing HF also played a mediating role, but because their β values were not completely isotropic, the proportion of mediating effect could not be calculated. Therefore, this article offers a novel perspective on the genetic pathogenesis of HF and aims to establish a foundation for future precision prevention and treatment strategies.

Triacylglycerol (50:1) levels, triacylglycerol (52:2) levels, and triacylglycerol (53:3) levels are all composed of 3 fatty acids and glycerol, just that their respective fatty acids differ in the number of carbon atoms and double bonds they contain, but they all belong to the triglyceride group. Triglycerides play a crucial role in energy storage and cellular metabolism within biological systems.^[34]^ When excessive accumulation of triglyceride occurs in the human body, it can lead to obesity and is associated with cardiovascular and metabolic diseases.^[35,36]^ While the heart is a high-energy consumption and high-activity organ,^[37]^ under normal physiological conditions, it mainly relies on the breakdown of myocardial triglycerides into fatty acids to provide energy for cardiac contraction, but impaired fatty acid metabolism can also lead to cardiac dysfunction. During the synthesis and decomposition of triglycerides, diglycerides, and ceramides related to lipid toxicity are also produced.^[38]^ Studies have confirmed that excessive triglyceride accumulation in the myocardium can lead to lipotoxic cardiomyopathy and cardiac steatosis.^[39]^ In a prospective study from Gothenburg, it was clearly stated that triglycerides are risk factors for HF,^[40]^ which aligns closely with our findings. This study also reveals that HLA-DR+ CD4+ AC mediates the association between triacylglycerol (50:1) levels and the risk of developing HF, and the risk of HF increases with increasing HLA-DR+ CD4+ AC. HLA-DR+ CD4+ AC is an activated T cell type, and HLA-DR is a part of the major histocompatibility complex class II molecules, which are essential for the activation of lymphocytes and the coordination of adaptive immune responses, and are involved in the process of antigen presentation to CD4+ T cells.^[41]^ In conjunction with the studies of Laroumanie et al^[42]^ and Nevers et al,^[43]^ persistently activated CD+ T cells affect the cardiac structure, whether under pressure overload or myocardial ischemic conditions, leading to compensatory left ventricular hypertrophy, fibrosis, remodeling, and ultimately HF.^[44]^ CD4+ T cells have also been found to be associated with HF in mouse models. In a mouse model of ischemic HF, the overall expansion and activation of CD4+ T cells is dominated by Th2 and Th17 subsets, leading to cardiac fibrosis, remodeling, and heart injury.^[45]^ In contrast, in a mouse model of HF resulting from pressure overload due to narrowing of the thoracic aorta, cardiac fibrosis and cardiac dysfunction are primarily influenced by Th1 cells.^[46]^ HLA-DR++ monocytes represent a subset of leukocytes characterized by high expression of HLA-DR. These monocytes are typically found in intermediate monocytes, with the ability to proliferate and activate T cells as well as antigen presentation. It has been observed that intermediate monocytes are associated with ST-segment elevation myocardial infarction, atherosclerosis, and other diseases.^[47]^ Although specific studies between HLA-DR++ monocyte %leukocyte and HF are currently lacking, its role in HF deserves further investigation.

Phosphatidylcholine (14:0_16:0) levels, phosphatidylcholine (16:0_20:1) levels, phosphatidylcholine (O-16:1_20:3) levels, and phosphatidylcholine (14:0_18:1) levels are all phosphatidylcholines, which have different biological functions and metabolic processes due to the different chain lengths, sn-positions, and carbon-carbon double-bond (C=C) positions on the fatty acid chains of each of them.^[48,49]^ Some studies have pointed out that disturbed phosphatidylcholine metabolism disrupts myocardial metabolism and cell signaling,^[50]^ especially in HF, the metabolism of phosphatidylcholine may be related to changes in myocardial lipid homeostasis.^[51]^ In Rong’s study, it was pointed out that phosphatidylcholine (20:0/18:4), phosphatidylcholine (20:4/20:0), phosphatidylcholine (40:4), phosphatidylcholine (20:4/18:0), phosphatidylcholine (C34:4), and phosphatidylcholine (36:5) were elevated in cases of HF, while phosphatidylcholine (32_0), phosphatidylcholine (C34:4), and phosphatidylcholine (36:5) decreased.^[52]^ Thus, it also confirms that different isoforms have different biological functions. In this context, we focus on the mediating role of CD45 on granulocytes in phosphatidylcholine (14:0_18:1) levels and the risk of developing HF. Phosphatidylcholine is an indispensable phospholipid in mammalian cell membranes, which directly supports immune cell function by participating in the structure and proliferation of immune cells. Studies have showed that phospholipids from different dietary sources have different roles depending on the fatty acids and head groups, for example, the consumption of soy-derived phosphatidylcholine has been shown to effectively reduce the inflammatory response of arthritis and similar inflammatory processes in mouse models, and it has also been found to be effective in lowering cholesterol levels, while the consumption of marine phospholipid source foods has been shown to inhibit chemically induced growth of colon cancer in vitro.^[53]^ Studies have also noted that high homocysteine concentrations are associated with an increased risk of CVD,^[54]^ while phosphatidylcholine can effectively reduce homocysteine levels, thereby reducing the risk of CVD.^[55]^ In a prospective cohort study, a higher intake of phosphatidylcholine was associated with a lower risk of type 2 diabetes.^[56]^ Therefore, we can speculate that some phosphatidylcholine may protect heart function by lowering cholesterol levels, homocysteine levels, and the risk of type 2 diabetes. Of course, further exploratory studies are needed to confirm the exact mechanism. This study also clarified that the risk of developing HF increases with the increase of CD45 in granulocytes, a granulocyte that expresses CD45, a receptor-like protein tyrosine phosphatase that is expressed in all leukocytes and is essential for the activation and proliferation of T cells.^[57]^ Granulocytes mainly include neutrophils and eosinophils; the neutrophils are the most numerous type of leukocytes, which play an important role in the inflammatory response. In the study by Martini et al, it was pointed out that neutrophils are strongly associated with the development of HF.^[58]^ A positive correlation between neutrophil levels and the risk of developing HF was confirmed in the CALIBER cohort study.^[59]^ In a prospective cohort study of acute coronary syndrome treated with percutaneous coronary intervention, it was noted that eosinophil levels were positively associated with the risk of developing HF.^[60]^ Thus, the present MR findings are highly consistent with those of previous studies. We hope to assist in further studies in the future, as well as provide inspiration for the development of precision medicine in this field.

This study utilized a large sample size and the latest GWAS data for MR analysis. A variety of methods were used for sensitivity analyses, yielding robust and reliable results. However, there are still some limitations: first, although HLA-DR+ CD4+ AC-mediated triacylglycerol (50:1) levels were found to affect HF, the proportion of the mediating effect was 5.77%, and there may be other mediators that need to be further investigated; second, the significance thresholds for plasma lipids and immune cells were set at *P* < 5 × 10^−6^, which may have some confounding factors; finally, the analyzed populations were all European populations, and whether they are suitable for other populations needs to be verified.

In conclusion, the present study utilized a bidirectional, two-sample, two-step MR study to show that there was a causal relationship between 7 plasma lipids and HF and that 4 immune cells mediated the causal relationship between lipids and HF, among which HLA-DR+ CD4+ AC-mediated triacylglycerol (50:1) levels affected HF with a mediating effect ratio of 5.77%. These findings underscore that plasma lipids and immune cells are potential targets in the pathogenesis of HF, offering a new direction for the development of targeted therapies for HF in the future.

## Acknowledgments

We are grateful to the institutions for making the GWAS summary data publicly available, and grateful to all the researchers and participants who contributed to those studies.

## Author contributions

**Conceptualization:** Zonglian Liu, Rongchuan Yue.

**Data curation:** Zonglian Liu, Yangyang Cui, Lijuan Xiong, Shikang Li, Lang Zeng, Linqin Du, Kun Wang.

**Methodology:** Zonglian Liu, Yangyang Cui, Shikang Li, Lang Zeng.

**Software:** Zonglian Liu.

**Validation:** Zonglian Liu.

**Writing – original draft:** Zonglian Liu, Eugene Kwaku.

**Visualization:** Linqin Du, Kun Wang.

**Writing – review & editing:** Jiankang Zheng, Dalin Luo, Gang Zeng, Rongchuan Yue.

**Funding acquisition:** Rongchuan Yue.

**Supervision:** Rongchuan Yue.

**Figure:**
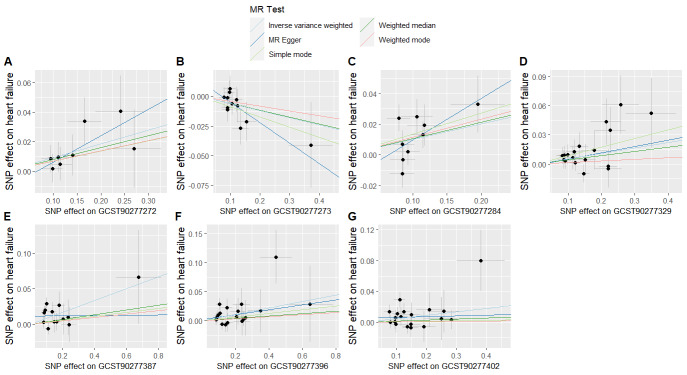






**Figure:**
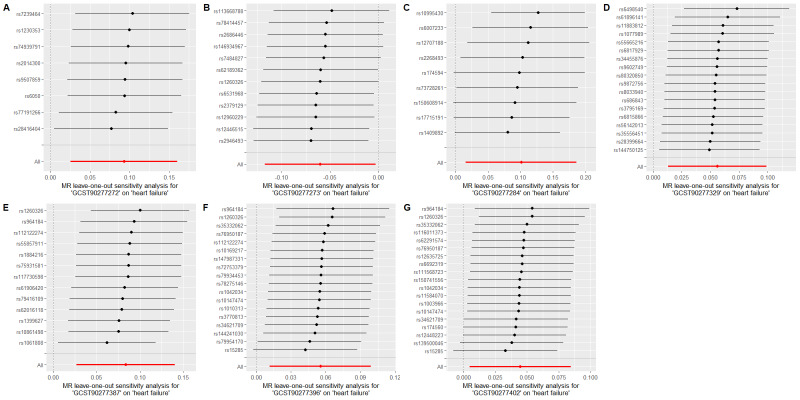


**Figure:**
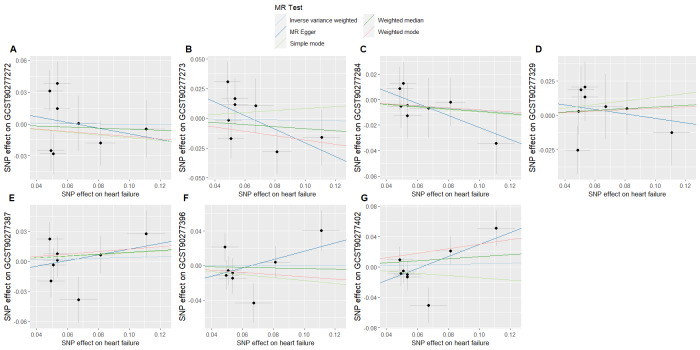






**Figure:**