# Supplementary material for: Immune cell-mediated effects of plasma lipids on heart failure: A two-step, two-sample Mendelian randomization study
Source: Medicine (Baltimore). 2026 May 29;105(22):e49074. doi: 10.1097/MD.0000000000049074 (PMC13225585; doi:10.1097/MD.0000000000049074)
Supplement: Supplementary file 1 [file medi-105-e49074-s002.docx]

**Table 1.**　Results of heterogeneity analysis between plasma lipids and heart failure

| Exposure factor | MR Egger | | IVW | | *I^2^* |
| --- | --- | --- | --- | --- | --- |
|  | *Q* | *Q_pval* | *Q* | *Q_pval* |  |
| Phosphatidylcholine (14:0_16:0) levels | 2.242 | 0.896 | 2.911 | 0.893 | 0 |
| Phosphatidylcholine (14:0_18:1) levels | 4.777 | 0.906 | 6.006 | 0.873 | 0 |
| Phosphatidylcholine (16:0_20:1) levels | 12.039 | 0.099 | 13.380 | 0.099 | 40.2% |
| Phosphatidylcholine (O-16:1_20:3) levels | 13.460 | 0.639 | 13.486 | 0.703 | 0 |
| Triacylglycerol (50:1) levels | 11.863 | 0.374 | 13.452 | 0.337 | 10.8% |
| Triacylglycerol (52:2) levels | 15.760 | 0.470 | 15.837 | 0.535 | 0 |
| Triacylglycerol (53:3) levels | 15.585 | 0.553 | 16.109 | 0.585 | 0 |
