# Supplementary material for: Immune cell-mediated effects of plasma lipids on heart failure: A two-step, two-sample Mendelian randomization study
Source: Medicine (Baltimore). 2026 May 29;105(22):e49074. doi: 10.1097/MD.0000000000049074 (PMC13225585; doi:10.1097/MD.0000000000049074)
Supplement: Supplementary file 2 [file medi-105-e49074-s003.docx]

**Table 2.**　Results of pleiotropic analysis between plasma lipids and heart failure

| Exposure factor | MR-Egger | | MR-PRESSO | |
| --- | --- | --- | --- | --- |
|  | intercept | pval | MR pval | Global Test P value |
| Phosphatidylcholine (14:0_16:0) levels | -0.012 | 0.445 | 0.004 | 0.890 |
| Phosphatidylcholine (14:0_18:1) levels | 0.013 | 0.293 | 0.018 | 0.878 |
| Phosphatidylcholine (16:0_20:1) levels | -0.017 | 0.406 | 0.048 | 0.147 |
| Phosphatidylcholine (O-16:1_20:3) levels | -0.001 | 0.874 | 0.011 | 0.683 |
| Triacylglycerol (50:1) levels | 0.011 | 0.250 | 0.014 | 0.347 |
| Triacylglycerol (52:2) levels | 0.002 | 0.786 | 0.020 | 0.523 |
| Triacylglycerol (53:3) levels | 0.005 | 0.479 | 0.033 | 0.563 |
