# Supplementary material for: Immune cell-mediated effects of plasma lipids on heart failure: A two-step, two-sample Mendelian randomization study
Source: Medicine (Baltimore). 2026 May 29;105(22):e49074. doi: 10.1097/MD.0000000000049074 (PMC13225585; doi:10.1097/MD.0000000000049074)
Supplement: Supplementary file 3 [file medi-105-e49074-s006.docx]

**Table 3.**　Results of heterogeneity analysis between heart failure and plasma lipids

| outcome | MR Egger | | IVW | | *I^2^* |
| --- | --- | --- | --- | --- | --- |
|  | *Q* | *Q_pval* | *Q* | *Q_pval* |  |
| Phosphatidylcholine (14:0_16:0) levels | 10.824 | 0.094 | 11.281 | 0.127 | 37.9% |
| Phosphatidylcholine (14:0_18:1) levels | 6.040 | 0.419 | 8.566 | 0.285 | 18.3% |
| Phosphatidylcholine (16:0_20:1) levels | 1.743 | 0.942 | 2.905 | 0.894 | 0 |
| Phosphatidylcholine (O-16:1_20:3) levels | 4.921 | 0.554 | 5.277 | 0.626 | 0 |
| Triacylglycerol (50:1) levels | 7.013 | 0.320 | 7.514 | 0.377 | 6.8% |
| Triacylglycerol (52:2) levels | 7.771 | 0.255 | 9.682 | 0.207 | 27.7% |
| Triacylglycerol (53:3) levels | 7.630 | 0.266 | 12.156 | 0.096 | 42.4% |
