# Supplementary material for: Immune cell-mediated effects of plasma lipids on heart failure: A two-step, two-sample Mendelian randomization study
Source: Medicine (Baltimore). 2026 May 29;105(22):e49074. doi: 10.1097/MD.0000000000049074 (PMC13225585; doi:10.1097/MD.0000000000049074)
Supplement: Supplementary file 4 [file medi-105-e49074-s007.docx]

**Table 4.**　Results of pleiotropic analysis between heart failure and plasma lipids

| outcome | MR-Egger | | MR-PRESSO | |
| --- | --- | --- | --- | --- |
|  | intercept | pval | MR pval | Global Test P value |
| Phosphatidylcholine (14:0_16:0) levels | 0.018 | 0.633 | 0.995 | 0.166 |
| Phosphatidylcholine (14:0_18:1) levels | 0.037 | 0.164 | 0.890 | 0.300 |
| Phosphatidylcholine (16:0_20:1) levels | 0.025 | 0.322 | 0.239 | 0.874 |
| Phosphatidylcholine (O-16:1_20:3) levels | 0.014 | 0.572 | 0.051 | 0.642 |
| Triacylglycerol (50:1) levels | -0.016 | 0.537 | 0.734 | 0.404 |
| Triacylglycerol (52:2) levels | -0.032 | 0.270 | 1.000 | 0.232 |
| Triacylglycerol (53:3) levels | -0.049 | 0.108 | 0.777 | 0.104 |
