# Supplementary figures and images for: Immune cell-mediated effects of plasma lipids on heart failure: A two-step, two-sample Mendelian randomization study
Source: Medicine (Baltimore). 2026 May 29;105(22):e49074. doi: 10.1097/MD.0000000000049074 (PMC13225585; doi:10.1097/MD.0000000000049074)

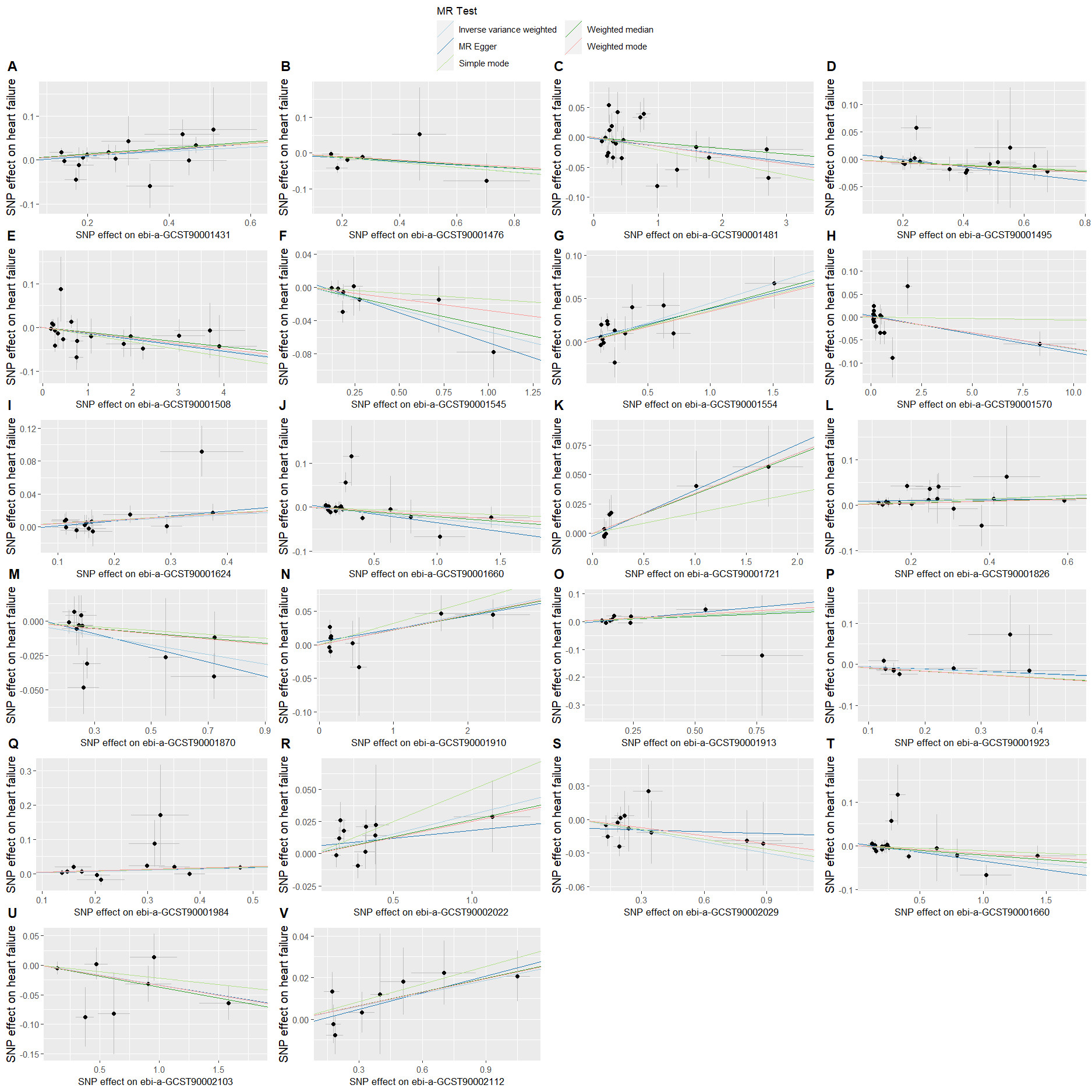

Supplement: Supplementary file 5 [file medi-105-e49074-s009.jpg]

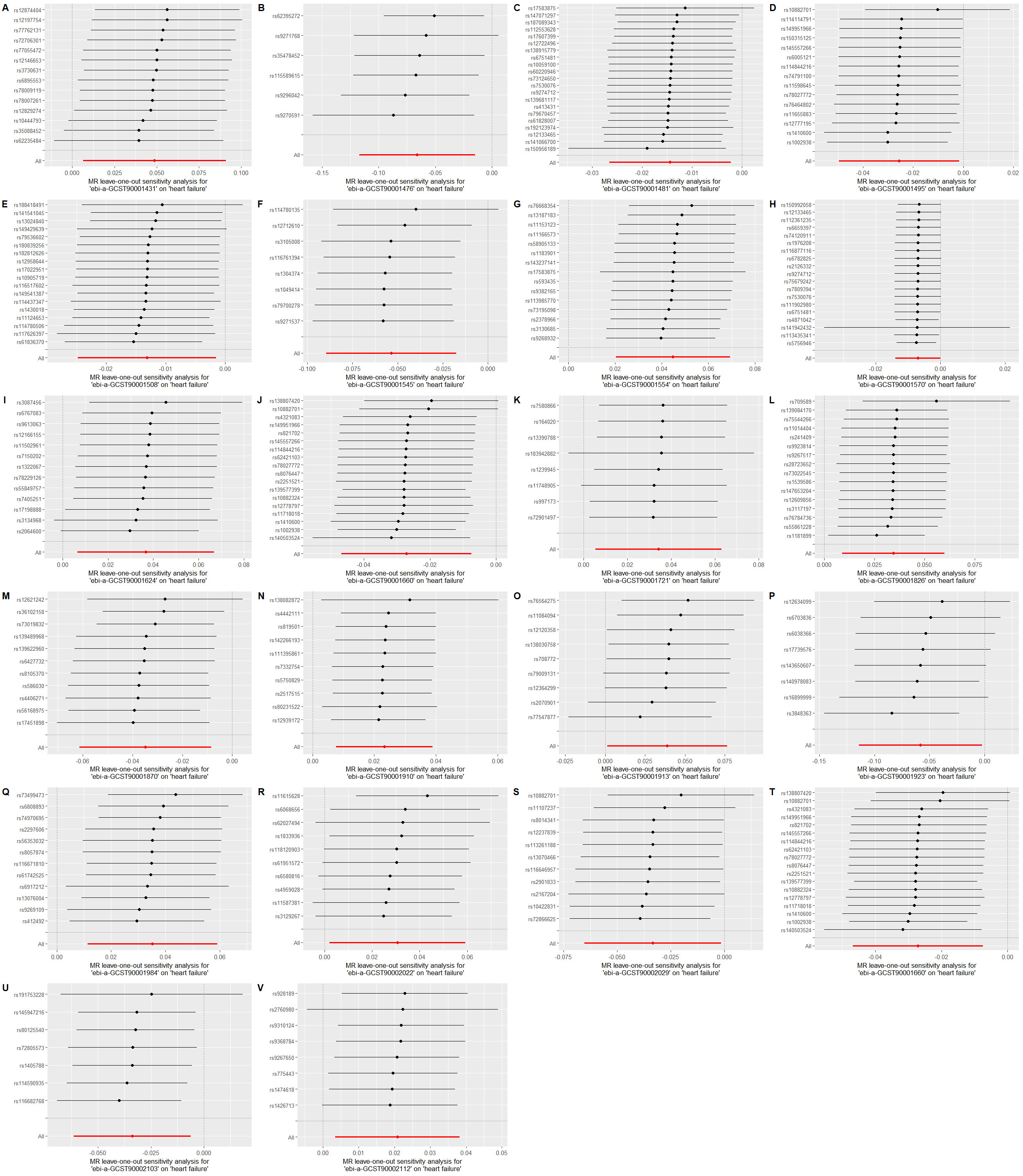

Supplement: Supplementary file 8 [file medi-105-e49074-s012.jpg]
