# Supplementary material for: Immune cell-mediated effects of plasma lipids on heart failure: A two-step, two-sample Mendelian randomization study
Source: Medicine (Baltimore). 2026 May 29;105(22):e49074. doi: 10.1097/MD.0000000000049074 (PMC13225585; doi:10.1097/MD.0000000000049074)
Supplement: Supplementary file 6 [file medi-105-e49074-s010.docx]

**Table 5.**　Results of heterogeneity analysis between immune cells and heart failure

| Exposure factor | MR Egger | | IVW | | *I^2^* |
| --- | --- | --- | --- | --- | --- |
|  | *Q* | *Q_pval* | *Q* | *Q_pval* |  |
| IgD+ CD38- %lymphocyte | 12.268 | 0.424 | 12.515 | 0.486 | 0 |
| HLA-DR++ monocyte %leukocyte | 7.148 | 0.128 | 7.193 | 0.207 | 30.5% |
| Resting Treg % CD4 Treg | 28.181 | 0.080 | 28.363 | 0.101 | 29.5% |
| CD39+ secreting Treg AC | 10.592 | 0.645 | 12.277 | 0.584 | 0 |
| CD25hi CD45RA+ CD4 not Treg %CD4+ | 19.071 | 0.265 | 19.077 | 0.324 | 10.9% |
| TD CD4+ AC | 3.968 | 0.681 | 4.530 | 0.717 | 0 |
| EM CD8br AC | 15.668 | 0.268 | 16.166 | 0.303 | 13.4% |
| EM DN (CD4-CD8-) %DN | 21.839 | 0.191 | 22.758 | 0.200 | 20.9% |
| HLA-DR+ CD4+ AC | 9.651 | 0.562 | 10.153 | 0.603 | 0 |
| CD39+ CD4+ AC | 19.520 | 0.243 | 20.888 | 0.231 | 18.6% |
| CD19 on CD20- | 2.275 | 0.893 | 2.453 | 0.931 | 0 |
| IgD on unsw mem | 15.497 | 0.345 | 17.573 | 0.286 | 14.6% |
| CD34 on HSC | 12.343 | 0.195 | 12.834 | 0.233 | 22.1% |
| CD45 on B cell | 8.645 | 0.373 | 9.395 | 0.402 | 4.2% |
| CD45 on granulocyte | 5.950 | 0.546 | 6.904 | 0.547 | 0 |
| CD127 on CD45RA- CD4 not Treg | 6.809 | 0.339 | 6.810 | 0.449 | 0 |
| HLA-DR on CD14- CD16+ monocyte | 13.537 | 0.195 | 13.558 | 0.258 | 18.9% |
| CD4 on CD4+ | 9.133 | 0.331 | 9.795 | 0.367 | 8.1% |
| CD39 on CD39+ CD8br | 7.813 | 0.553 | 8.911 | 0.541 | 0 |
| CD45 on Mo MDSC | 1.508 | 0.912 | 2.011 | 0.919 | 0 |
| CD45RA on CD39+ resting Treg | 4.907 | 0.427 | 4.909 | 0.556 | 0 |
| HLA-DR on CD33- HLA DR+ | 3.396 | 0.758 | 3.666 | 0.817 | 0 |
