# Supplementary material for: Immune cell-mediated effects of plasma lipids on heart failure: A two-step, two-sample Mendelian randomization study
Source: Medicine (Baltimore). 2026 May 29;105(22):e49074. doi: 10.1097/MD.0000000000049074 (PMC13225585; doi:10.1097/MD.0000000000049074)
Supplement: Supplementary file 7 [file medi-105-e49074-s011.docx]

**Table 6.**　Results of pleiotropic analysis between immune cells and heart failure

| Exposure factor | MR-Egger | | MR-PRESSO | |
| --- | --- | --- | --- | --- |
|  | intercept | pval | MR pval | Global Test P value |
| IgD+ CD38- %lymphocyte | -0.005 | 0.632 | 0.038 | 0.547 |
| HLA-DR++ monocyte %leukocyte | -0.004 | 0.881 | 0.052 | 0.319 |
| Resting Treg % CD4 Treg | -0.002 | 0.730 | 0.031 | 0.167 |
| CD39+ secreting Treg AC | 0.013 | 0.217 | 0.042 | 0.531 |
| CD25hi CD45RA+ CD4 not Treg %CD4+ | ＜0.001 | 0.945 | 0.040 | 0.417 |
| TD CD4+ AC | 0.006 | 0.482 | 0.008 | 0.736 |
| EM CD8br AC | 0.003 | 0.532 | 0.003 | 0.357 |
| EM DN (CD4-CD8-) %DN | 0.003 | 0.409 | 0.063 | 0.601 |
| HLA-DR+ CD4+ AC | -0.005 | 0.494 | 0.023 | 0.656 |
| CD39+ CD4+ AC | 0.005 | 0.305 | 0.015 | 0.259 |
| CD19 on CD20- | -0.002 | 0.687 | 0.006 | 0.973 |
| IgD on unsw mem | 0.008 | 0.192 | 0.018 | 0.305 |
| CD34 on HSC | 0.006 | 0.564 | 0.027 | 0.298 |
| CD45 on B cell | 0.004 | 0.429 | 0.017 | 0.571 |
| CD45 on granulocyte | -0.009 | 0.361 | 0.061 | 0.492 |
| CD127 on CD45RA- CD4 not Treg | -0.589×10^-3^ | 0.977 | 0.077 | 0.445 |
| HLA-DR on CD14- CD16+ monocyte | -0.001 | 0.902 | 0.015 | 0.305 |
| CD4 on CD4+ | 0.006 | 0.468 | 0.065 | 0.385 |
| CD39 on CD39+ CD8br | -0.008 | 0.322 | 0.055 | 0.579 |
| CD45 on Mo MDSC | -0.011 | 0.510 | 0.010 | 0.922 |
| CD45RA on CD39+ resting Treg | -0.450×10^-3^ | 0.971 | 0.038 | 0.651 |
| HLA-DR on CD33- HLA-DR+ | -0.003 | 0.622 | 0.014 | 0.894 |
