# Supplementary material for: Immune cell-mediated effects of plasma lipids on heart failure: A two-step, two-sample Mendelian randomization study
Source: Medicine (Baltimore). 2026 May 29;105(22):e49074. doi: 10.1097/MD.0000000000049074 (PMC13225585; doi:10.1097/MD.0000000000049074)
Supplement: Supplementary file 9 [file medi-105-e49074-s013.docx]

**Table 7.**  Heterogeneity analysis results of mediated analysis

| exposure factor | outcome factor | MR Egger | | IVW | | *I^2^* |
| --- | --- | --- | --- | --- | --- | --- |
|  |  | *Q* | *Q_pval* | *Q* | *Q_pval* |  |
| Phosphatidylcholine (14:0_18:1) levels | heart failure | 4.777 | 0.906 | 6.006 | 0.873 | 0 |
| Phosphatidylcholine (14:0_18:1) levels | CD45 on granulocyte | 4.081 | 0.944 | 5.612 | 0.898 | 0 |
| CD45 on granulocyte | heart failure | 5.950 | 0.546 | 6.904 | 0.547 | 0 |
| Triacylglycerol (50:1) levels | heart failure | 11.863 | 0.374 | 13.452 | 0.337 | 10.8% |
| Triacylglycerol (50:1) levels | HLA DR+ CD4+ AC | 7.112 | 0.790 | 7.120 | 0.850 | 0 |
| HLA-DR+ CD4+ AC | heart failure | 9.651 | 0.562 | 10.153 | 0.603 | 0 |
| Triacylglycerol (52:2) levels | heart failure | 15.760 | 0.470 | 15.837 | 0.535 | 0 |
| Triacylglycerol (52:2) levels | TD CD4+ AC | 6.797 | 0.977 | 6.934 | 0.984 | 0 |
| TD CD4+ AC | heart failure | 3.968 | 0.681 | 4.530 | 0.717 | 0 |
| Triacylglycerol (53:3) levels | heart failure | 15.585 | 0.553 | 16.109 | 0.585 | 0 |
| Triacylglycerol (53:3) levels | HLA DR++ monocyte %leukocyte | 7.793 | 0.971 | 7.889 | 0.980 | 0 |
| HLA-DR++ monocyte %leukocyte | heart failure | 7.148 | 0.128 | 7.193 | 0.207 | 30.5% |
