# Supplementary material for: Immune cell-mediated effects of plasma lipids on heart failure: A two-step, two-sample Mendelian randomization study
Source: Medicine (Baltimore). 2026 May 29;105(22):e49074. doi: 10.1097/MD.0000000000049074 (PMC13225585; doi:10.1097/MD.0000000000049074)
Supplement: Supplementary file 10 [file medi-105-e49074-s014.docx]

**Table 8.**  Results of pleiotropic analysis of mediated analysis

| exposure factor | outcome factor | MR-Egger | | MR-PRESSO | |
| --- | --- | --- | --- | --- | --- |
|  |  | intercept | pval | MR pval | Global Test  P value |
| Phosphatidylcholine (14:0_18:1) levels | heart failure | 0.013 | 0.293 | 0.018 | 0.882 |
| Phosphatidylcholine (14:0_18:1) levels | CD45 on granulocyte | -0.051 | 0.244 | 0.008 | 0.894 |
| CD45 on granulocyte | heart failure | -0.009 | 0.361 | 0.061 | 0.519 |
| Triacylglycerol (50:1) levels | heart failure | 0.011 | 0.250 | 0.014 | 0.339 |
| Triacylglycerol (50:1) levels | HLA-DR+ CD4+ AC | -0.002 | 0.930 | 0.018 | 0.851 |
| HLA DR+ CD4+ AC | heart failure | -0.005 | 0.494 | 0.023 | 0.661 |
| Triacylglycerol (52:2) levels | heart failure | 0.002 | 0.786 | 0.020 | 0.559 |
| Triacylglycerol (52:2) levels | TD CD4+ AC | 0.009 | 0.717 | 0.002 | 0.979 |
| TD CD4+ AC | heart failure | 0.006 | 0.482 | 0.008 | 0.759 |
| Triacylglycerol (53:3) levels | heart failure | 0.005 | 0.479 | 0.033 | 0.611 |
| Triacylglycerol (53:3) levels | HLA DR++ monocyte %leukocyte | 0.007 | 0.761 | 0.001 | 0.975 |
| HLA-DR++ monocyte %leukocyte | heart failure | -0.004 | 0.881 | 0.052 | 0.292 |
